# Pruritus Ani

**Published:** 1907-05

**Authors:** 


### PRURITUS ANI.

T. Chittenden Hill says that by far the most important causes of pruritus ani are superficial ulcerations or abrasions of the anal canal. The lesion is very constant and should always be looked for. The writer then discusses various other conditions associated frequently with this affection, among which are catarrhal diseases, hemorrhoids, and polyps. Appropriate treatment must be applied to the unnatural skin before much amelioration of the itching can be expected. After defecation the patient should cleanse thoroughly the anal region, preferably with absorbent cotton which has been wrung dry of some antiseptic solution. An anal pad held in place by a T bandage will protect the parts from friction and irritation of the discharge. Nitrate if silver and citrine ointment are excellent applications in most cases. For delicate skins the following ointment may be substituted for the citrine: red oxide of mercury three drachms, Venice turpentine one ounce, lanolin three ounces.—Medical Record, December 22, 1906.

